# Genome-Wide Identification, and In-Silico Expression Analysis of YABBY Gene Family in Response to Biotic and Abiotic Stresses in Potato (*Solanum tuberosum*)

**DOI:** 10.3390/genes14040824

**Published:** 2023-03-29

**Authors:** Hafiz Sabah-Ud-Din Mazhar, Muhammad Shafiq, Haider Ali, Muhammad Ashfaq, Alia Anwar, Javaria Tabassum, Qurban Ali, Ghulam Jilani, Muhammad Awais, Ravi Sahu, Muhammad Arshad Javed

**Affiliations:** 1 Department of Plant Breeding and Genetics, Faculty of Agricultural Sciences, University of the Punjab, Lahore 54590, Pakistan; hafiz.sabahuddin@outlook.com (H.S.-U.-D.M.);; 2 Department of Horticulture, Faculty of Agricultural Sciences, University of the Punjab, Lahore 54590, Pakistan; 3 School of Biosciences, University of Birmingham, Edgbaston, Birmingham B15 2TT, UK; 4 Department of Agronomy, University of Agriculture, Faisalabad 38040, Pakistan; 5 Geography Earth and Environmental Sciences, University of Birmingham, Edgbaston, Birmingham B15 2TT, UK

**Keywords:** expression pattern, genome-wide analysis, potato, transcription factor, *YABBY* gene family

## Abstract

*YABBY* is among the specific transcription factor (TF) gene family in plants and plays an important role in the development of the leaves and floral organs. Its specific roles include lateral organ development, the establishment of dorsoventral polarity, and response to abiotic stress. Potato is an important crop worldwide and *YABBY* genes are not still identified and characterized in potato. So, little has been known about *YABBY* genes in potato until now. This study was carried out to perform genome-wide analysis, which will provide an in-depth analysis about the role of *YABBY* genes in potato. There have been seven *StYAB* genes identified, which are found to be located on seven different chromosomes. Through multiple sequence analyses, it has been predicted that the *YABBY* domain was present in all seven genes while the C2-C2 domain was found to be absent only in *StYAB2*. With the help of cis-element analysis, the involvement of *StYAB* genes in light, stress developmental, and hormonal responsiveness has been found. Furthermore, expression analysis from RNA-seq data of different potato organs indicated that all *StYAB* genes have a role in the vegetative growth of the potato plant. In addition to this, RNA-seq data also identified *StYAB3*, *StYAB5*, and *StYAB7 genes* showing expression during cadmium, and drought stress, while *StYAB6* was highly expressed during a viral attack. Moreover, during the attack of *Phytophthora infestans* on a potato plant *StYAB3*, *StYAB5*, *StYAB6*, and *StYAB7* showed high expression. This study provides significant knowledge about the *StYAB* gene structures and functions, which can later be used for gene cloning, and functional analysis; this information may be utilized by molecular biologists and plant breeders for the development of new potato lines.

## 1. Introduction

*YABBY* transcription factors (TF) are plant specific and belong to a subfamily of zinc finger protein. These gene family members have two regions, N-terminal zinc finger region (C2-C2), and the C-terminal *YABBY* region; both domains contain highly conserved amino acids (AA) that are specifically involved in DNA binding [1]. The *YABBY* gene family encodes six genes in *Arabidopsis thaliana*, which are further categorized into five subfamilies as: FILAMENTOUS FLOWER (*FIL*)/*YAB1,* CRABS CLAW (*CRC*), INNER NO OUTER (*INO*)*, YAB2*, and *YAB5*. The *YAB2* gene played a role in flower development, *CRC* genes have a role in carpel polarity and the development of the nectary, and *INO* genes deal with the outer integument development of ovule, while *YAB5*/*YAB3* are associated with vegetative growth of leaves and cotyledons [2,3]. The *YABBY* TF also plays an important role in the lateral growth [4] and expansion of leaves [5], the flower development [6], the carpel development [7], the development of ovules [8], the development of veins in leaves [9], shattering of the seeds in cereals [10], the plant response to abiotic stress [11], and fruit ripening [12,13]. The *YABBY* gene has been found to be affected by hormones such as auxin, abscisic acid (ABA), and gibberellins [11,14]. 

Based on the crucial roles of *YABBY* genes in plant development, the genome-wide characterization and expression of *YABBY* genes have been studied in many plants (both angiosperms and gymnosperms). Until now, *YABBY* genes have been identified and characterized in different plant species, and the number of genes varied from 6 to 23. The number of *YABBY* genes reported are 9 in *Ananas comosus* [6], 7 in *Vitis vinifera* [12], 11 in *Cucurbita moschata*, 12 in *Cucurbita maxima*, 11 in *Cucurbita pepo* [15], 16 in *Phyllostachys edulis* [2], 11 in *Brassica rapa*, 11 in *Brassica oleracea*, 12 in *Brassica nigra*, 19 in *Brassica juncea*, 21 in *Brassica napus* [16,17], 17 in *Glycine max* [11], 12 in *Gossypium arboreum*, 12 in *Gossypium raimondii*, 23 in *Gossypium hirsutum* [3], 21 in *Triticum aestivum* [18], 8 in *Phaseolus vulgaris* (Common bean) [19], 9 in *Solanum lycopersicum* [20], 8 in *Oryza sativa* [21,22], 13 in *Zea mays* [23], and 9 in *Lactuca sativa* [24].

The expression patterns of *YABBY* genes in the vegetative tissues of monocot species are quite diverse. For instance, maize *ZYB9* and *ZYB14*, which are *FIL*/*YAB3*-like genes, are expressed adaxially and are believed to regulate lateral outgrowth [25]. In rice, a well-studied monocot species, *YABBY* gene functions have been extensively explored. The homolog of *CRC*, *OsDL*, has been found to influence both the leaves and flower development [26]. Additionally, during rice domestication, the *YABBY* genes *OsSh1* and *ObSh3* were found to be required for seed shattering. *OsYAB1* has been identified as a regulator of meristem development and of the maintenance of the stamens and carpels [27]. *OsYAB3 (TOB3)*, *OsYAB4 (TOB2)*, and *OsYAB5 (TOB1)* are enriched in lateral organ primordia and play a crucial role in rice spikelet development [28,29]. These findings demonstrate that *YABBY* genes have diverse functions in plant growth and development, with each subgroup exhibiting both differentiated and conserved functions. In core eudicots, the vegetative *YABBY* genes have been found to participate in various aspects of leaf development, and some of these genes also regulate shoot apical meristem development and phyllotaxy. Although *YABBY* genes are only expressed in abaxial regions, in *Zea mays* adaxial-specific expression is observed during the leaf primordia initiation [30]. Moreover, the *CRC* gene in eudicots is limited to the nectaries and carpels while its ortholog in monocots is involved in the development of the leaf midrib [31]. Therefore, the evolutionary history of *YABBY* genes seems to coincide with the emergence of leaves in seed plants [27], highlighting the need to comprehend the inter-species and intra-species relationships of *YABBY* genes. Potato (*Solanum tuberosum*) is an essential component of the human diet and its consumption is increasing day by day [32]. Potato is the world’s most important crop followed by maize, rice, and wheat. This vital crop has a very high potential and nutritional value [33]. *S. tuberosum* is a rich source of minerals, proteins, fats, vitamins, and energy. Potatoes are not only limited to food sources but also used drastically in industrial products such as potato chips, potato starch and powder flakes and pellets, and certain other value-added products [34]. Potato crop often faces the issue of low yields, because it is a root crop and is obtained as a result of vegetative growth [35]. As *YABBY* genes played a crucial role in vegetative development and also played a role in abiotic stresses, many of yield losses can be minimized if *YABBY* genes have been identified and functionally characterized in potato crops. Despite the immense importance of potato as a food crop, no significant research regarding its *YABBY* TFs has been reported until now. The objective of the study was the identification of the genes belonging to the *YABBY* TF family in potato genome and inter-operating their functions with the help of RNA-seq data through various bioinformatics tools. The results of genome-wide identification and characterization will be utilized for functional analysis and cloning of the members of this gene family.

## 2. Materials and Methods

### 2.1. Database Search and Retrieval of Sequences

The amino acid (AA) sequence of the *YABBY* domain was retrieved from Pfam database (accessed on 2 December 2021) and the *YABBY* domain (PF04690) in these sequences was identified. This domain (PF04690) was used for the identification of *StYAB* genes in potato genome database at Phytozome v13 (https://phytozome-next.jgi.doe.gov, accessed on 2 December 2021 using BLAST-P (Protein-basic local alignment search tool) program. The retrieved sequences of AA were verified on NCBI CDD (Conserved Domain Database) (http://www.ncbi.nlm.nih.gov/Structure/cdd/wrpsb.cgi) accessed on 2 December 2021 with the default parameters. The proteins lacking a real *YABBY* domain (PF04690) (https://pfam.xfam.org/family/PF04690) accessed on 2 December 2021 in their sequences were excluded.

### 2.2. Determination of Physio-Chemical Properties of YABBY Proteins

The ProtParam tool (accessed on 2 December 2021) (http://web.expasy.org/protparam/) was used to predict the protein length (AA residues), molecular weight, and pI value of *YABBY* proteins. The information on gene IDs, chromosomal positions, sequences of gene, and protein was taken from Phytozome. These *YABBY* encoding genes were renamed based on the order of their physical position. The subcellular localization of *StYAB genes* were predicted with the help of WoLF PSORT (accessed on 2 December 2021) (https://wolfpsort.hgc.jp/).

### 2.3. Gene Structure Analysis

For the prediction of the gene structure, genomic sequences, and 1000 bp upstream promoter, the sequences of *StYAB* genes were retrieved from Phytozome v13 (accessed on 2 December 2021). Furthermore, the gff3 file of the potato genome was retrieved from the PLAZA genome database (https://bioinformatics.psb.ugent.be/plaza/) accessed on 2 December 2021. These sequences were further used to visualize the gene structure using the gene structure display server (GSDS v2.0) (accessed on 3 December 2021) (http://gsds.cbi.pku.edu.cn/) to find the intron-exon arrangement, PlantCare database (accessed on 4 December 2021) (http://bioinformatics.psb.ugent.be/webtools/plantcare/html/) for cis-regulatory elements detection, and Multiple EM for motif elicitation (MEME) program (http://meme.nbcr.net/meme/) accessed on 4 December 2021 for motif recognition.

### 2.4. Comparative Phylogenetic Analysis

Phylogenetic analysis was performed between the genomes of *S. tuberosum*, *A. thaliana*, *S. lycopersicum*, *C. sativus*, and *C. maxima*. The AA sequences of *YABBY* proteins were aligned using MUSCLE [36]. These protein sequences were used to generate the phylogenetic tree in MEGA 11 software using the neighbor-joining (NJ) method, with a bootstrapping value set at 1000 replications. It was further modified using iTOL (https://itol.embl.de/) accessed on 5 December 2021.

### 2.5. Gene Duplication and Synteny Analysis

The divergence time of *StYAB* genes was estimated with the help of Ka/Ks values. The protein sequence alignment was made using MUSCLE and the number of Ka/Ks substitution rates were determined using Tbtools v 1.108. Default parameters were used as described in the package manual. Ka/Ks ratios of paralogous genes were calculated in order to estimate the molecular evolution rate of each gene pair. The time of divergence (DT) was estimated by putting the Ks value in T = Ks/2λ equation, where λ is equal to 2.6 × 10^−9^ [37]. MCScanX v 1.0 (Multiple Collinearity Scan toolkit) was adopted to analyze the gene duplication events, with the default parameters. To find the synteny relationship between the paralogous genes of potato and orthologous genes of *Arabidopsis*, tomato, and chili, syntenic and dual syntenic maps were constructed using Tbtools [38].

### 2.6. Gene Ontology Enrichment Analysis

Gene ontology (GO) term enrichment analysis was performed to further validate the functions of *StYAB* genes using GO annotations [31]. Uniprot (Online database) (accessed on 8 December 2021) (https://www.uniprot.org/) was used to find out the molecular functions of genes as well as the various biological processes. Furthermore, an online web tool ShinyGo v0.741 (accessed on 9 December 2021) (http://bioinformatics.sdstate.edu/go/) was used for the analysis to better understand the role of *StYAB* genes in potatoes.

### 2.7. Expression Analysis

Expression data of all seven *StYAB* genes were taken from different plant organs (unfertilized leaves, floral buds, axial regulator, developing tubers, petioles, and stems) and under different biological stresses (viral attack, *Phytophthora infestans* attack on leaves, cadmium stress, drought stress, and meristem dormancy). Expression analysis of *StYAB* genes was performed using previously generated RNA-seq data. Data related to the growth stages and the behavior of the different organs under various biotic and abiotic stresses was retrieved from three databases, i.e., NCBI gene expression omnibus (GEO) (accessed on 15 December 2021), expression atlas (accessed on 17 December 2021), and expressed sequence tags (EST) (accessed on 20 December 2021). For the expression profiling, reads per kilobases per million mapped read (RPKM) values from the RNA-seq data were log_2_ transformed [39]. The data for expression in different organs and stresses were downloaded from previously published papers [40,41,42,43,44].

### 2.8. MiRNA Analysis

The micro-RNA (miRNA) sequences related to *StYAB* genes in potato were identified from psRNATarget (accessed on 25 December 2021) (https://www.zhaolab.org/psRNATarget/) using the CDS sequences of all the *StYAB* genes with the default parameters. The putative functions of the identified miRNA were retrieved from previously performed in vitro and in vivo experiments.

## 3. Results

### 3.1. Identification of the YABBY Genes in Potato

There were seven *StYAB* genes identified in potato. The *StYAB* genes encoding the protein’s length vary from 142 to 219 AA and the molecular weight varies from 16.15737 to 24.48169 kD, with *StYAB2* being the smallest and *StYAB3* being the longest protein (Table 1). In total, 33 out 56 AAs in the conserved *YABBY* domain were found to be 100% conserved in all the *YABBY* domain sequences of potato (Figure S1), while the remaining 23 AA were found to be variable in all the *StYAB* proteins. Furthermore, sub-cellular localization of *StYAB* genes showed that *StYAB7*, *StYAB3*, *StYAB4,* and *StYAB2*, were localized in a nucleus, while *StYAB6* and *StYAB1* were probably localized in the extracellular structures. The *StYAB5* genes were localized in the chloroplast (Table 2 and Figure S2).

### 3.2. Gene Structures and Recognition of Conserved Cis-Elements and Motifs

**Figure 1:**
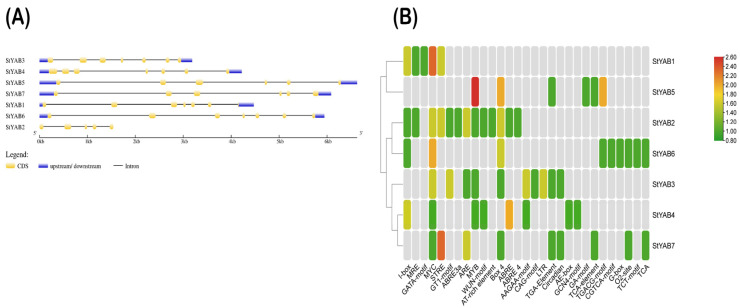
Predicted gene structures and cis-regulatory elements in *StYAB.* (**A**) Predicted gene structure of seven *StYAB* genes. The yellow regions show the exon regions, black lines show the introns, while the blue portions show the upstream/downstream sequences. (**B**) *Cis*-regulatory elements in putative *StYAB* promoters, which are associated with different plant developmental processes.

The gene structure prediction showed that four genes contain seven exons and six introns (57.14%), two genes had six exons and five introns (28.57%), and one gene had five exons and four introns (14.28%) (Table 1 and Figure 1A).

In total, 62 cis-elements were found in the *StYAB* genes (Figure 1A). These include light-responsive (24), stress-responsive (6), development- and metabolism-responsive (18), and hormone-responsive (14) elements. Overall, 38% of the elements were found to be responsive to light, indicating that *StYAB* genes play an important role in the light stress response. Six cis-elements were found to be related to a stress response, which predicts its role in stress-related activity. Moreover, a total of 18 cis-elements were found to be related to the development and metabolism of plants (Table S1). It makes up 29% of the cis-elements and it hints towards the role of *StYAB* genes in the growth and development of potato plants. Furthermore, three hormones related to cis-elements were identified, including Auxin, MeJA, and ABA, signifying the potential targets to study the role of hormones under stress conditions. It has been found that most hormone-related regions are dispersed in all seven genes. This signifies the role of these genes in the hormonal response (Figure 1B and Table S1).

**Figure 2:**
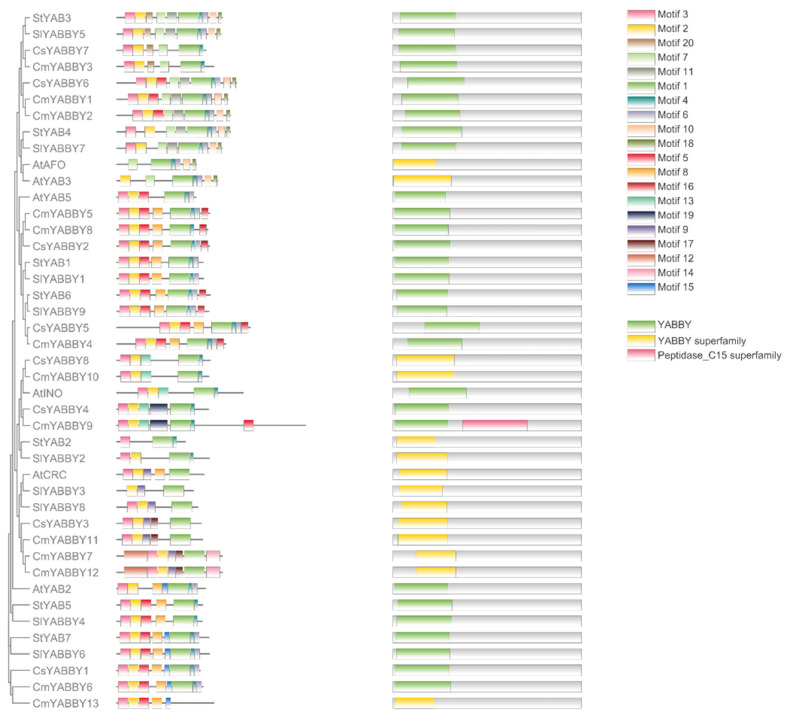
The distribution of 15 motifs on seven StYAB proteins. The motifs were found in potato by using MEME version 4.9.0 and inter-linking it with a phylogenetic tree to develop a good understanding of their association. The bars represent motifs with different color codes for the different types of motifs.

The distribution of 15 motifs on 7 YABBY proteins of potato by using MEME version 4.9.0 and interlinking it with phylogenetic tree to develop a good understanding of their association. The bars represent motifs with different color codes for different types of motifs (Figure 2).

### 3.3. Phylogenetic Analysis of the YABBY Gene Family

**Figure 3:**
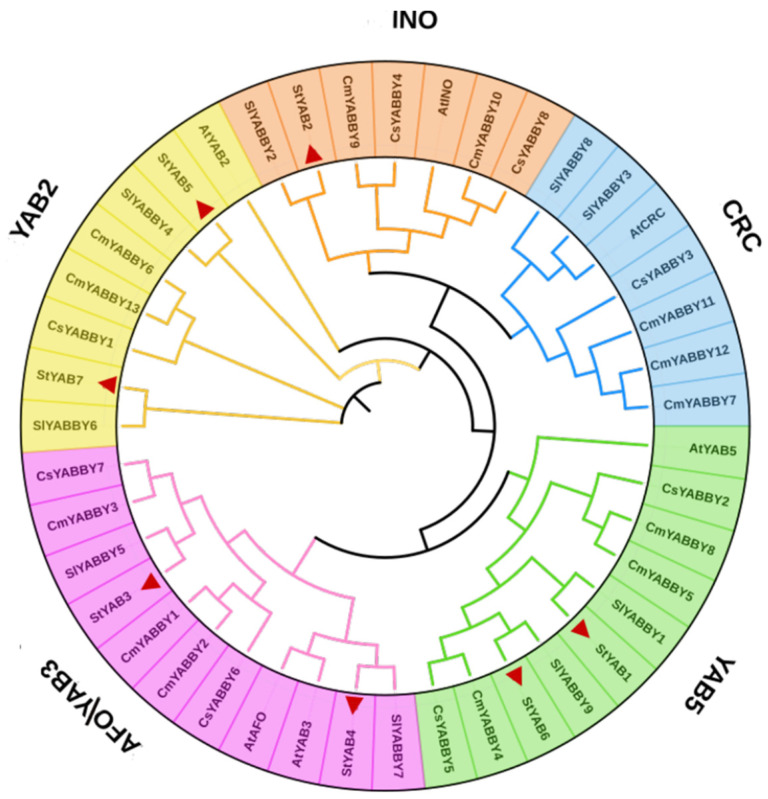
Phylogenetic relationship among the *YABBY* genes of *S. tuberosum*, *A. thaliana*, *S. lycopersicum*, *C. sativus*, and *C. maxima* was studied. *S. tuberosum* genes are marked with a red triangle. The evolutionary history was inferred using the NJ method with 1000 bootstrap replications. This analysis involved 43 *YABBY* genes. The evolutionary analyses were conducted in MEGA 11.

The results of the phylogenetic analysis depicted that *StYAB* genes can be categorized into five subgroups as in Arabidopsis, i.e., *INO*, *CRC*, *YAB5*, *AFO/YAB3*, and *YAB2.* Among these subgroups, *AFO/YAB3* is the biggest group containing 11 genes, *YAB5* is second with 10 genes, *YAB2* is third with 8 genes, *INO* is fourth with 7 genes, and *CRC* is the smallest with 7 genes. No potato gene was reported in the *CRC* group (Figure S3 and Table S2). It was also found that all the *StYAB* genes showed the close similarity with *S. lycopersicum* in all the identified groups (Figure 3). It is inferred from the phylogenetic analysis that *StYAB* genes might be directly evolved from *SlYAB* genes.

### 3.4. StYAB Gene Location on Chromosomes and Duplication Assessment

The chromosomal localization of the *StYAB* genes predicted that these genes were distributed on different chromosomes. *StYAB1*, *StYAB2*, *StYAB3*, *StYAB4*, *StYAB5*, *StYAB6*, and *StYAB7* were present on chromosomes 7, 5, 8, 1, 11, 12, and 6 respectively (Table 1; Figure S4).

**Figure 4:**
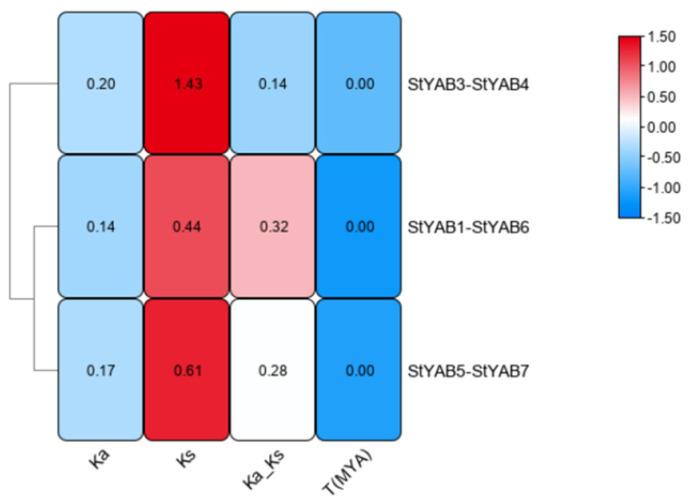
The time of gene duplication was estimated for the different paralogous pairs of the potato *StYAB* genes on the basis of Ks and Ka values. The analyses were conducted using Tbtools v 1.108. Ka/Ks represents the ratio of nonsynonymous (Ka) versus synonymous (Ks) mutations.

This estimated ratio was 0.14 between *StYAB3* and *StYAB4,* 0.28 between *StYAB5* and *StYAB7*, and 0.32 between *StYAB1* and *StYAB6*. The estimated date of duplication was 275.3137046 million years ago (MYA) in *StYAB3–StYAB4*, 117.7436777 (MYA) in *StYAB5–StYAB7*, and 85.22345807 (MYA) in *StYAB1–StYAB6* (Figure 4; Table S3).

**Figure 5:**
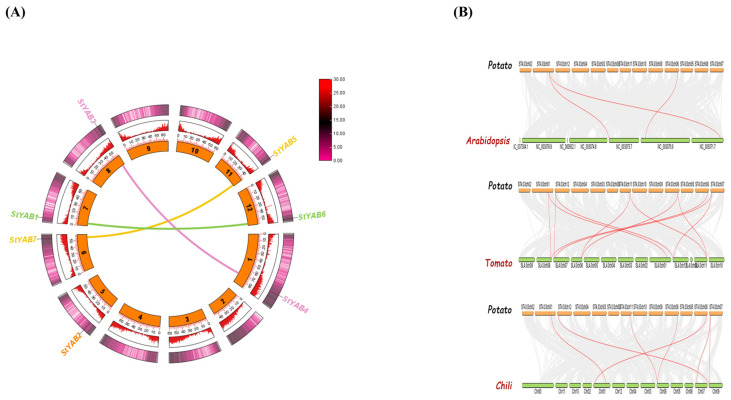
(**A**) The genome-wide synteny analysis of *StYAB* genes showing the dominance of segmental duplication and rare occurrence of tandem duplication. The joining lines show the duplicated *StYAB* genes in the genome. (**B**) Dual synteny analysis of potato-Arabidopsis, potato-tomato, and potato-chili. The orange bars represent the chromosomes of potato while the green bars represent the chromosomes of Arabidopsis, tomato, and chili, respectively. The red lines show the duplicated genes in the respective genomes.

The syntenic analysis showed that *StYAb1, StYAB3,* and *StYAB7* are the paralogs of *StYAB6, StYAB4,* and *StYAB5,* respectively, while the *StYAB2* gene happened to be a singleton as no duplicate has been found (Figure 5A). The dual synteny analysis showed that Arabidopsis had 3 duplicated genes. In between potato and tomato, 11 duplicated genes were found, while 7 genes were found duplicated in between potato and chili (Figure 5B).

### 3.5. Enrichment Analysis and Ortholog Identification

**Figure 6:**
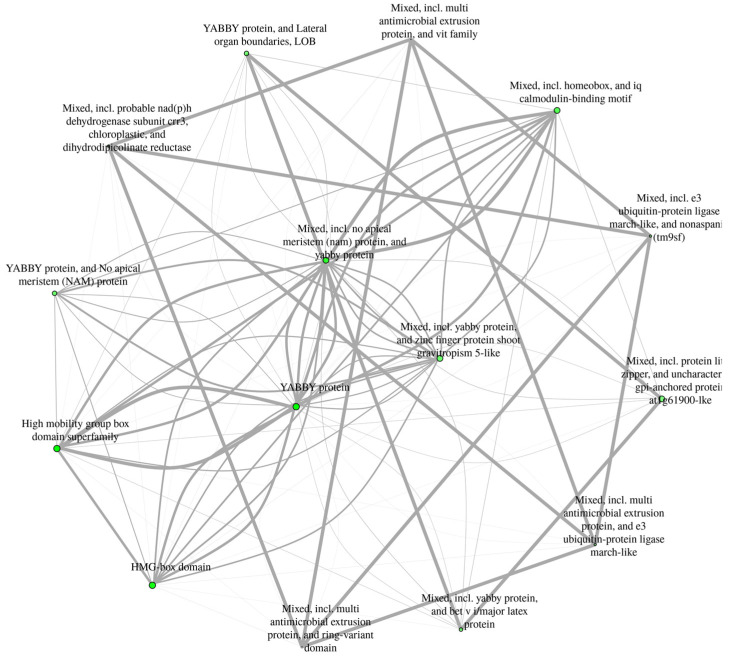
Static network enrichment graph, showing the network of *StYAB* gene functions. The darker nodes are more the significantly enriched gene sets. The bigger nodes represent the larger gene sets. The thicker edges represent more overlapped genes.

The GO analysis showed that the *StYAB* genes are involved in the diverse cellular functions and biological processes. All seven *StYAB* genes are involved in the abaxial cell fate specification and cellular development. Moreover, it was found that *StYAB* genes further participate in leaf, shoot, and meristem development (Table 2, Figure 6 and Figure S5).

### 3.6. Expression Analysis of StYAB Gene

**Figure 7:**
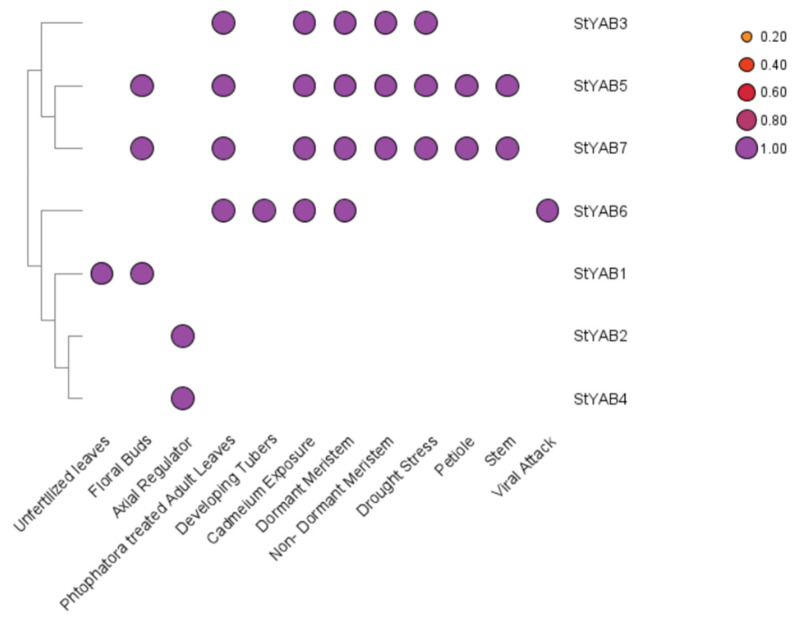
Heat map shows the expression profile of the *StYAB* genes in different organs and under different biological stresses of potato.

The results of the expression analysis showed that *StYAB* genes are highly expressed in different organs such as unfertilized leaves, floral buds, developing tubers, dormant meristems, petioles, and stems [40,41,42]. The differential expression of specific *StYAB* are shown in Figure 7. These results showed that the *StYAB* genes were involved in the vegetative development of potato plants. The different *StYAB* genes also showed their expression under different stress conditions (viral attack, *Phytophthora infestans* attack on leaves, cadmium stress, drought stress, and meristem dormancy) [40,41,42,43,44]. The result of RNA-seq data showed that *StYAB3*, *StYAB5*, and *StYAB7* were differentially expressed in response to cadmium stress and drought stress. The *StYAB6* was highly expressed when there was any viral attack. Moreover, the *StYAB3*, *StYAB5*, *StYAB6,* and *StYAB7* were highly expressed under *Phytophthora infestans* infection. These results suggested that the *StYAB* genes played a significant role under the different abiotic and biotic stress conditions (Figure 7 and Figures S6–S11).

### 3.7. MiRNA Targets in POTATO

A total of 10 miRNA sequences targeting 5 *StYAB* genes (*StYAB3*, *StYAB4*, *StYAB5*, *StYAB6*, and *StYAB7*) were detected. The expectation value observed was from 4 to 5 and the minimum length of the miRNA was 21 bp to a maximum of 24 bp. The miRNA sequences targeting *StYAB1* and *StYAB2* were not found (Table 3 and Table S4).

## 4. Discussion

Potato (*Solanum tuberosum* L.) is a highly important crop species worldwide, with global production exceeding 388 million metric tons per year, it is considered a promising crop for reducing human hunger and poverty due to its high yield potential and nutritional properties. Despite being predominantly grown by resource-poor farmers in developing countries, potato crops are susceptible to yield losses caused by various abiotic and biotic stresses. Transcription factors (TFs) are important regulatory elements that enable plants to respond to external environmental stresses, with families such as *NAC*, *bZIP*, *MYB*, *YABBY*, *NF-YB*, and *AP2* being particularly relevant in minimizing the yield losses [45,46]. Among these families, the *YABBY* TF family has received a lot of attention in plant research, especially in relation to its involvement in early embryonic development, lateral organ development, the establishment of the near-distal axis polarity of leaves, and plant development and stress response [47].

While *YABBY* genes have been extensively studied in dicotyledonous plants such as Arabidopsis, relatively little is known about monocot *YABBY* genes, including those of potato. Thus, a genome-wide study was conducted to identify and characterize the *StYAB* genes in potato. The study identified seven *StYAB* genes in total and found that their gene structure varied in terms of the number of exons and introns, potentially leading to functional variability. Furthermore, we observed different subcellular localizations for the *StYAB* genes, with four localized in the nucleus, two in extracellular structures, and one in the chloroplast. An analysis of the *StYAB* gene promoters revealed the presence of various cis-regulatory elements related to plant growth and development, light, and stress response, further supporting the idea that these genes are involved in different vegetative growth and have a role in mitigating different biotic and abiotic stress conditions.

To better understand the possible functions of *StYAB* genes in the Solanaceae family, expression patterns of different genes were analyzed under different stress conditions. It was found that *StYAB1*, *StYAB3*, *StYAB2*, and *StYAB5* exhibited similar levels of expression to their homologs in Arabidopsis and rice, indicating functional conservation. Additionally, while only one homolog of the *INO* group was observed in *S. tuberosum*, we found that *StYAB2*, the homolog of the *INO* group, was expressed in processes related to axial regulation. These results suggest that *StYAB* genes have diverse functions in plant growth and development, and may play a role in mitigating the different stress conditions. However, further extensive studies are necessary to better understand the specific functions of *StYAB* genes.

The *YABBY* TF family is a subgroup of the zinc finger protein superfamily, and its members contain two structural domains: the zinc-finger domain and *YABBY* domain [48]. The amino acids in these domains are linked with DNA specificity and are highly conserved [49]. While Arabidopsis, tomato, and other dicotyledonous species have five sub-groups, including *CRC*, *FIL/YAB3*, *INO*, *YAB2*, and *YAB5*, monocotyledon rice has only four sub-groups, lacking *YAB5*. During evolution, *YABBY* TFs in monocots, dicots, and different subpopulations have undergone functional differentiation. In this study, *StYAB* genes were classified into five groups as per in *A. thaliana*, i.e., *INO*, *YAB2*, *YAB5*, *AFO*, and *CRC*. However, no member of the *StYAB* gene family was detected in the *CRC* group.

Gene duplication is a common evolutionary event that occurs when an organism’s genome acquires an additional copy of a pre-existing gene. This can occur through various mechanisms, such as unequal crossing over, retro-transposition, and whole-genome duplication. Gene duplication plays a critical role in evolution, as it creates opportunities for functional divergence and innovation. The duplicated genes can acquire new functions through various mechanisms, including sub-functionalization and neo-functionalization, leading to an increase in the complexity and diversity of organisms [50].

Synteny analysis is a powerful tool used to study gene duplication events and their evolutionary consequences. It involves the comparison of the order and arrangement of genes on different chromosomes, or within a genome. By identifying the syntenic regions, researchers can detect the gene duplications and infer the evolutionary history of the gene families [56]. The synteny analysis can also reveal the mechanisms of gene duplication, such as segmental or tandem duplication, and provide insights into the functional diversification of the gene families.

This can be predicted from their chromosome location. This means genes that are present on the same chromosome can possibly be the result of tandem duplication, whereas those genes that are located on different chromosomes might possibly be the result of segmented duplication. In the case of *StYAB* genes, segmented duplication was predominantly observed. 

Interestingly, a similar pattern of segmented duplication was observed in the *YABBY* gene family of *Oryza sativa*. This finding is consistent with the results of *StYAB* gene synteny analysis in potato, as well as in other plant species such as *C. arietinum* and *Olea europaea*. These results highlight the importance of gene duplication events in shaping the evolution of plant genomes and the diversification of gene families. Further investigation of the functional divergence and neofunctionalization of *YABBY* genes in these plant species could shed light on their role in the development and evolution of plant structures. It was further confirmed with the help of synteny analysis (Figure 5A and Figure S5). The same results were observed in the case of *C. arietinum* [51] and *Olea europaea* [52]. Gene duplication is the main driver of genome expansion in species. Variations in the gene family members across different eukaryotic species might be because of the process of evolution [53]. Moreover, the dual synteny blocks of potato were constructed with three crops, i.e., Arabidopsis, tomato, and chili, separately to determine the connection between them. The dual syntenic block of potato and Arabidopsis genomes predicted that two genes in chromosome 1 and one gene of chromosome 6 have duplicated genes in Arabidopsis as it was shown by joining threads between the respective chromosomes (Figure 5b). The dual syntenic block between the potato and tomato genome predicted that both species share 11 duplicated genes from different chromosomes as shown by joining threads (Figure 5b). Lastly, the dual synteny of potato and chili showed that they share seven similar genes on different chromosomes, as shown in (Figure 5b).

MicroRNAs (miRNAs) are important regulatory entities of plants. They have a role in the regulation of almost all biological processes of plants, such as plant growth and development, during biotic and abiotic stress. They are highly conserved and are very specific in function. The Stu-miR390-3p member of family miR390 was detected, targeting *StYAB7*. This family has a role in promoting lateral growth [54], which means that *StYAB7* has a role in primary growth and inhibits lateral growth. Four members of the miR5303 family, stu-miR5303g, stu-miR5303h, stu-miR5303i, and stu-miR5303j, were detected targeting our *StYAB4* gene, and play important roles in long RNA development during tuber sprouting [55], which depicts that *StYAB4* has a role that somewhat inhibits tuber sprouting in potato. One member from the miR172 family was detected, stu-miR172d-5p, targeting our gene *StYAB3* [57]. This miRNA (stu-miR172d-5p) promotes nodulation in potato by inhibiting *StYAB3*. One member from the miR1886 family, i.e., stu-miR1886g-5p, was detected targeting two genes *StYAB4* and *StYAB7*. This miRNA has a role in plant growth and development [58]. Furthermore, two miRNA families, i.e., miR7992 and miR8037, with one member each detected in potato, targeted *StYAB6* and *StYAB5* respectively. miR7992-5p targets nuclear inclusion protein [59], while no role of miR8037 was found (Table 3).

## 5. Conclusions

This study identified seven *StYAB* genes in *Solanum tuberosum*. Based on structural analysis, it was found that *StYAB* genes have five to seven exons. Based on motif analysis and promoter analysis, it was found that cis-regulatory elements related to light, and developmental and hormonal responsiveness were present in the promoter of *StYAB* genes, which conferred their role in the vegetative development of potatoes. Apart from that, some cis-regulatory elements related to biotic and abiotic stress were also found. Along with the presence of cis-regulatory elements, expression analysis results demonstrated that *StYAB1*, *StYAB2*, and *StYAB4* had a role in the vegetative development of the potato plant. Additionally, *StYAB* genes showed expression during different abiotic and biotic stresses. *StYAB3*, *StYAB5*, *StYAB6*, and *StYAB7* showed expression during the *Phytophthora infestans* attack, while *StYAB6* also showed higher expression levels during the virus attack on potato plants. Furthermore, *StYAB3*, *StYAB5*, and *StYAB7* also showed expression during abiotic stresses, i.e., cadmium and drought stress. However, this in-silico study represents a comprehensive genome-wide knowledge of *StYAB* genes in potato, but further investigation such as gene cloning and functional analysis are required to validate these genes and their functional roles against the different physiological and biological processes.

## Figures and Tables

**Table 1:** Information about the discovered seven *YABBY* genes in potato genome. Accession number, chromosome number and location, gene direction, amino acid sequence length, molecular weight, isoelectric point (Pi value), no. of introns and exons.

*YABBY* Gene Name	Accession Number		Chromosome Number	Chromosome Location (bp)		Direction	No. of Amino Acids	Molecular Weight (kD)	Pi Value	No. of Introns	No. of Exons
	Phytozome V13 (6.1)	PLAZA Genome Database		Start	End						
*StYAB 1*	Soltu.DM.07G003610	PGSC0003DMT400079392	7	4307036	4311510	F	179	20.16703	8.77	6	7
*StYAB 2*	Soltu.DM.05G008980	PGSC0003DMT400072893	5	9003101	9004644	F	142	16.15737	8.25	4	5
*StYAB 3*	Soltu.DM.08G025380	PGSC0003DMT400012083	8	55208205	55211393	R	219	24.48169	7.22	6	7
*StYAB 4*	Soltu.DM.01G031660	PGSC0003DMT400066793	1	71511821	71516043	R	218	24.16953	7.23	6	7
*StYAB 5*	Soltu.DM.11G024670	PGSC0003DMT400007731	11	44742927	44749556	R	178	20.14184	9.24	5	6
*StYAB 6*	Soltu.DM.12G026680	PGSC0003DMT400007474	12	56595139	56601086	R	194	22.09788	8.17	6	7
*StYAB 7*	Soltu.DM.06G029310	PGSC0003DMT400015197	6	54473907	54479995	F	191	21.272	8.79	5	6

**Table 2:** Gene ontology enrichment analysis of *StYAB* genes their GO functions, sub-cellular localization signal genes expression, orthologs in arabidopsis, and their functions are presented in the table.

Gene ID	GO Function		Sub-Cellular Localization	Gene Expression	Ortholog in Arabidopsis		References
	Molecular Function	Biological Process			Gene Name	Function	
*StYAB1*	Abaxial cell fate specification	Leaf development	Extra chromosomal	Unfertilized leaves and floral buds	*YABBY* 5	Axial regulator	[45,46]
*StYAB2*	Cell fate commitment, floral bud determinacy, and style development	Leaf and flower development	Nucleus	Axial regulator	INO	Outer integument growth	[47]
*StYAB3*	Fruit development, polarity specification of abaxial-adaxial surface, regulation of shoot apical meristem, and leaf development	Leaf, flower, and fruit development	Nucleus	Phytophthora treated adult leaves, cadmium stress, dormant and non-dormant potato tubers, photoperiod effect, phloem associated cells	*YABBY*1/FIL	Axial regulator	[45]
*StYAB4*	Fruit development, polarity specification of abaxial-adaxial surface, regulation of shoot apical meristem, and leaf development	Leaf, flower, and fruit development	Nucleus	Axial regulator	*YABBY*3	Axial regulator	[47]
*StYAB5*	Abaxial cell fate commitment	Leaf development	Chloroplast	Floral buds, cadmium stress, dormant and non-dormant potato tubers, photoperiod effect, phloem associated cells	*YABBY*2	Putative axial regulator	[48,49]
*StYAB6*	Abaxial cell fate commitment	Leaf development	Extra chromosomal	Developing tubers, cadmium stress, dormant and non-dormant potato tubers, photoperiod effect, phloem associated cells	*YABBY*5	Axial regulator	[45,50]
*StYAB7*	Abaxial cell fate commitment	Leaf development	Nucleus	Floral buds, cadmium stress, dormant and non-dormant potato tubers, photoperiod effect, phloem associated cells	*YABBY*2	Putative axial regulator	[48,50]

**Table 3:** *StYAB* genes targeting putative miRNA functions along with their targeted genes.

Gene	Targeting miRNA	Function	Reference
*StYAB3*	Stu-miR172d-5p	Enhance nodulation	[51]
*StYAB4*	stu-miR5303g, stu-miR5303i, stu-miR5303h, stu-miR1886g-5p, stu-miR5303j,	Long Non-RNA development during tuber sprouting	[52]
*StYAB5*	stu-miR8037	No target site reported	[53]
*StYAB6*	stu-miR7992-5p	Targets nuclear inclusion protein	[54]
*StYAB7*	stu-miR390-3p, stu-miR1886g-5p	Promotes lateral growth	[55]

## Supplementary Materials

The following supporting information can be downloaded at: https://www.mdpi.com/article/10.3390/genes14040824/s1, Figure S1: The sequence logos are based on alignments of *StYAB* gnese; Figure S2: Heat Map representing sub-cellular localization of all seven *StYAB* genes; Figure S3: *YABBY* genes distribution chart representing, total number of *YABBY* genes from different species in 5 sub-groups based on phylogenetic analysis; Figure S4: Distribution of *StYAB* genes on potato chromosomes, lines predicting the possible gene duplication on different chromosomes; Figure S5: Fold Enrichment chart representing the overlapping *StYAB* genes functions; Figure S6: Heat map shows the expression profile of the *StYAB* genes in leaves and Roots of potato cultivar under different levels of Cadmium stress i.e., Control = 0 mg per kg, T1 = 1 mg per kg and T2 = 5 mg per kg); Figure S7: Heat map shows the expression profile of the *StYAB* genes in Dormant and Non- Dormant potato cultivar; Figure S8: Heat map shows the expression profile of the *StYAB* genes in leaves of four different potato cultivars under drought stress; Figure S9: Heat map shows the expression profile of the *StYAB* genes in petiole of potato cultivars under different photoperiod; Figure S10: Heat map shows the expression profile of the *StYAB* genes in phloem associated cells of petiole and lower stem of potato cultivar; Figure S11: Heat map shows the expression profile of the *StYAB* genes in tubers of two potato cultivars (Phytophthora Infestans resistant and susceptible) after different time of inoculating infection in both cultivars (0 h, 24 h, and 48 h); Table S1: Putative Cis-regulatory elements found in promoter regions of *StYAB* genes; Table S2: *StYAB* genes distribution among groups based on phylogenetic analysis; Table S3: Ka/Ks ratio duplicated gene pairs in potato; Table S4: miRNA targets prediction of *StYAB* genes. The miRNA data was downloaded from psRNATarget (Online Web tool).
