# Supplementary material for: Genome-Wide Identification, and In-Silico Expression Analysis of YABBY Gene Family in Response to Biotic and Abiotic Stresses in Potato (Solanum tuberosum)
Source: Genes (Basel). 2023 Mar 29;14(4):824. doi: 10.3390/genes14040824 (PMC10137784; doi:10.3390/genes14040824)

Table S1:

Putative Cis-regulatory elements found in promoter regions of *StYAB* genes.

Gene Id	Start-Seq	End-seq	Cis-Elements	Function
StYAB1	447	438	I-box	Part of light responsive element
StYAB1	682	675	MRE	MYB binding site involved in light responsive elements
StYAB1	449	439	GATA-motif	Part of light responsive element
StYAB1	89	95	MYC	Activates expression of proliferative genes
StYAB1	853	848	STRE	Stress related function
StYAB2	42	37	STRE	Stress related function
StYAB2	78	84	MYC	Activates expression of proliferative genes
StYAB2	173	179	GT1-motif	light responsive element
StYAB2	791	797	ABRE3a	Positive regulator of abiotic stress and ABA signaling
StYAB2	899	893	ARE	cis-acting regulatory element essential for the anaerobic induction
StYAB2	663	672	I-box	Part of light responsive element
StYAB2	680	674	MYB	Plant Development
StYAB2	461	470	WUN-motif	A defense and stress responsive element
StYAB2	234	244	AT-rich element	Binding site of AT-rich DNA binding protein (ATBP-1)
StYAB2	226	232	Box 4	Part of a conserved DNA module involved in light responsiveness
StYAB2	792	797	ABRE	Cis-acting element involved in the abscisic acid responsiveness
StYAB2	791	785	ABRE 4	Mediate ABA-dependent stress responses.
StYAB2	674	667	MRE	MYB binding site involved in light responsive elements
StYAB3	521	528	AAGAA-motif	cis-acting regulatory element
StYAB3	583	589	ARE	cis-acting regulatory element essential for the anaerobic

				induction
StYAB3	835	829	Box 4	part of a conserved DNA module involved in light responsiveness
StYAB3	763	753	CAG-motif	part of a light response element
StYAB3	640	646	GT1-motif	light responsive element
StYAB3	199	193	LTR	cis-acting element involved in low-temperature responsiveness
StYAB3	799	805	MYB	Plant Development
StYAB3	899	905	MYC	Activates expression of proliferative genes
StYAB3	727	721	TGA-Element	Auxin-responsive element
StYAB3	176	185	Circadian	cis-acting regulatory element involved in circadian control
StYAB4	565	572	AAGAA-motif	cis-acting regulatory element
StYAB4	531	537	ABRE	Cis-acting element involved in the abscisic acid responsiveness
StYAB4	953	961	AE-box	part of a module for light response
StYAB4	410	416	ARE	cis-acting regulatory element essential for the anaerobic induction
StYAB4	768	758	GATA-motif	Part of light responsive element
StYAB4	1100	1107	GCN4-motif	cis-regulatory element involved in endosperm expression
StYAB4	221	211	I-box	Part of light responsive element
StYAB4	576	582	MYB	Plant Development
StYAB4	1024	1018	MYC	Activates expression of proliferative genes
StYAB4	445	436	WUN-motif	A defense and stress responsive element
StYAB5	111	117	Box 4	Part of conserved DNA module involved in light responsiveness
StYAB5	895	887	GA-motif	Part of light responsive element

StYAB5	676	682	MYB	Plant Development
StYAB5	69	78	TCA element	cis-acting element involved in salicylic acid responsiveness
StYAB5	341	347	TGA-Element	Auxin-responsive element
StYAB5	481	486	TGACG-motif	cis-acting regulatory element involved in the MeJA-responsiveness
StYAB6	1	7	Box 4	Part of conserved DNA module involved in light responsiveness
StYAB6	343	338	CGTCA-motif	cis-acting regulatory element involved in the MeJA-responsiveness
StYAB6	766	760	G-box	light responsive element
StYAB6	520	511	I-box	light responsive element
StYAB6	376	370	MYC	Activates expression of proliferative genes
StYAB6	51	59	O2-site	cis-acting regulatory element involved in zein metabolism regulation
StYAB6	257	266	TCA	Salicylic acid responsiveness
StYAB6	739	745	TCT-motif	light responsive element
StYAB6	343	348	TGACG-motif	cis-acting regulatory element involved in MeJA-responsiveness
StYAB7	982	988	ARE	Anaerobic induction
StYAB7	807	801	Box 4	light responsive element
StYAB7	734	728	MYC	Activates expression of proliferative genes
StYAB7	474	463	O2-site	cis-acting regulatory element involved in zein metabolism regulation
StYAB7	164	169	STRE	Stress related function
StYAB7	863	853	TCA	Salicylic acid responsiveness
StYAB7	398	389	TCA element	Salicylic acid responsiveness
StYAB7	679	685	TGA-Element	Auxin-responsive element

StYAB7	375	384	Circadian	cis-acting regulatory element involved in circadian control

Table S2

StYAB genes distribution among groups based on phylogenetic analysis.

Groups	Potato		Arabidopsis		Tomato		Cultivated Squash		Cucumber	
	**NO. of *YABBY genes***	**Gene-id**	**NO. of *YABBY genes***	**Gene-id**	**NO. of *YABBY genes***	**Gene-id**	**NO. of *YABBY genes***	**Gene-id**	**NO. of *YABBY genes***	**Gene-id**
**INO**	1	*StYAB 2.*	1	*AtINO*	1	*SlYABBY 2*	2	*CmYABBY9, CmYABBY10*	2	*CsYABBY 4, CsYABBY 8*
**YAB2**	2	*StYAB 7, StYAB 5.*	1	*AtYAB 2*	2	*SlYABBY 6, SlYABBY 4*	2	*CmYABBY13, CmYABBY6*	1	*CsYABBY 1*
**AFO / YAB3**	2	*StYAB 4, StYAB 3.*	2	*AtAFO, AtYAB 3*	2	*SlYABBY 5, SlYABBY 7*	3	*CmYABBY1, CmYABBY2, CmYABBY3*	2	*CsYABBY 6, CsYABBY 7*
**YAB5**	2	*StYAB 1, StYAB 6.*	1	*AtYAB 5*	2	*SlYABBy1, SlYABBY 9*	3	*CmYABBY4, CmYABBY5, CmYABBY8*	2	*CsYABBY 2, CsYABBY 5*
**CRC**	0		1	*AtCRC*	2	*SlYABBY 3, SlYABBY 8*	3	*CmYABBY7, CmYABBY11, CmYABBY12*	1	*CsYABBY 3*

Table S3:

Ka/Ks ratio duplicated gene pairs in potato.

Gene Id	Ka	Ks	Ka/Ks	T(MYA)
**StYAB1-StYAB6**	0.140981	0.443162	0.318126	8.52235E-05
**StYAB3-StYAB4**	0.19983	1.431631	0.139582	0.000275314
**StYAB5-StYAB7**	0.168917	0.612267	0.275888	0.000117744

Table S4:

miRNA targets prediction of *StYAB* genes. The miRNA data was downloaded from psRNATarget (Online Web tool).

miRNA	Target Gene	E-value	Length	Target-start'	Target-end'	miRNA-sequence
**stu-miR390-3p**	*StYAB7*	4	21	485	505	CGCUAUCCAUCUUGAGUUUUA
**stu-miR5303g**	*StYAB4*	4	24	185	209	AUAUUUUUGAAGAGUC-UGAGCAAC
**stu-miR5303i**	*StYAB4*	4	24	185	209	AUAUUUUUGAAGAGUC-UGAGCAAC
**stu-miR172d-5p**	*StYAB3*	4.5	21	343	362	GGAGCAUCAUCAAGAUUCACA
**stu-miR5303h**	*StYAB4*	4.5	24	186	210	AACAUUUUUGAAGAGUC-UGAGCAA
**stu-miR1886g-5p**	*StYAB7*	5	24	448	471	GAGAUGAGAUCAAUGUUUGGACAU
**stu-miR1886g-5p**	*StYAB4*	5	24	681	703	GAGAUGAGAUCAAUGUUUGGACAU

**stu-miR5303j**	*StYAB4*	5	24	186	210	AAUAUUUUUGAAGAGUC-UGAGCAA
**stu-miR7992-5p**	*StYAB6*	5	24	540	563	UUUGACAAUGCACAUCUAGACACU
**stu-miR8037**	*StYAB5*	5	22	280	301	AUAAUUUGGAGGAAUAGGAACC

Figure S1:

The sequence logos are based on alignments of *StYAB* gnese. *YABBY* domain is highly conserved across all *StYAB genes* while C2-C2 domain is highly conserved in only six *YABBY* proteins (*StYAB1*, *StYAB3*, *StYAB4*, *StYAB5*, *StYAB6*, *StYAB7*). Black bars representing start and end point of C2-C2 type domain & YABBY domain. Single alphabet on the respective number representing the highly conserve nature of that respective amino acid in s all *StYAB* genes sequence.

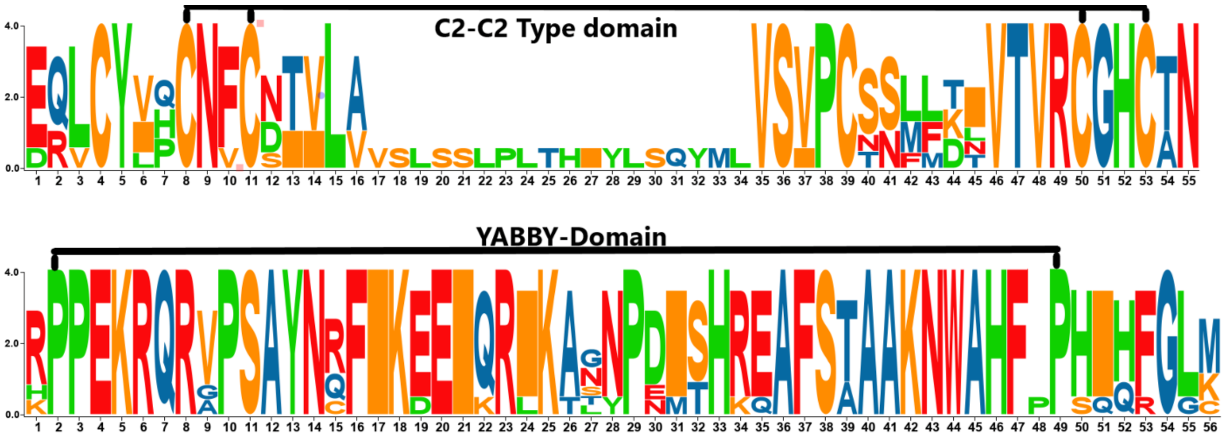

Figure S2:

Heat Map representing sub-cellular localization of all seven *StYAB* genes. Grey color represents absence of respective genes in that cell region. White and Red color represents minimum and maximum value of gene in that region.

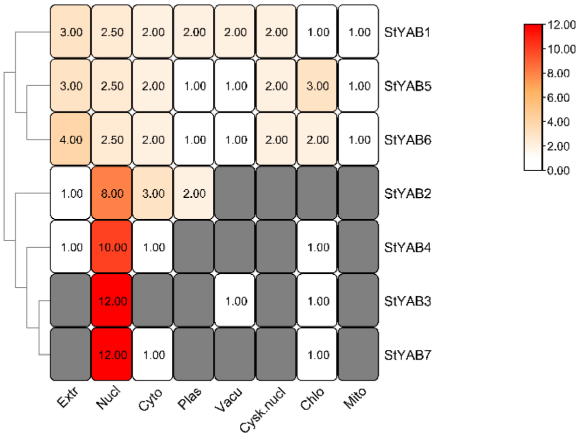

Figure S3:

YABBY genes distribution chart representing, total number of *YABBY* genes from different species in 5 sub-groups based on phylogenetic analysis.

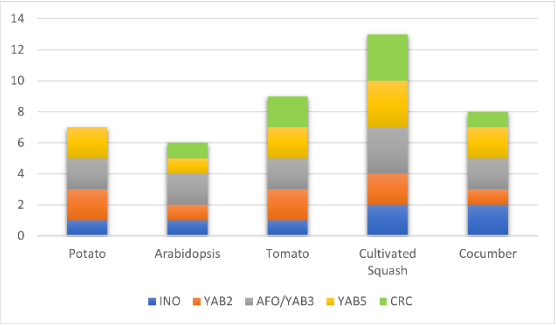

Figure S4:

Distribution of *StYAB* genes on potato chromosomes, lines predicting the possible gene duplication on different chromosomes.

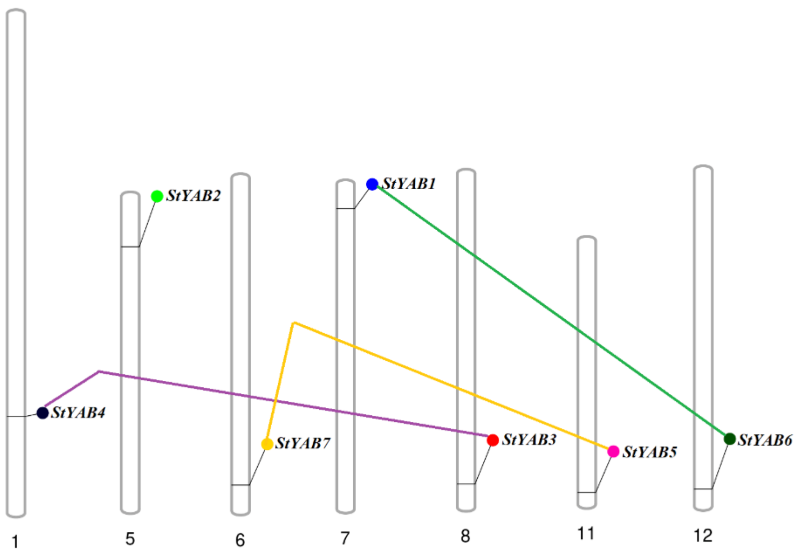

Figure S5:

Fold Enrichment chart representing the overlapping *StYAB* genes functions. Bigger lollipop tip size represents the more no. of genes involved in that process and vice versa for small tip size.

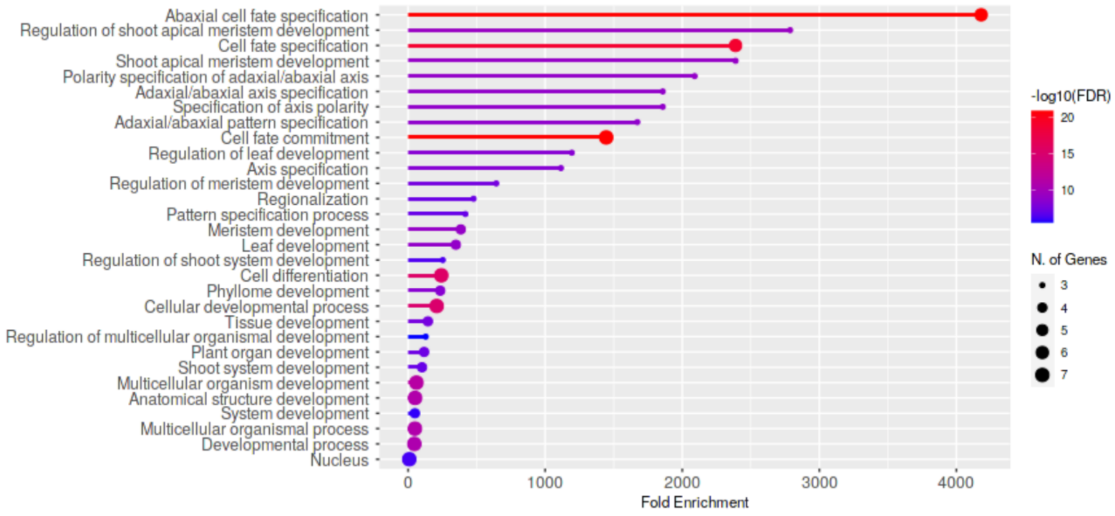

Figure S6:

Heat map shows the expression profile of the *StYAB* genes in leaves and Roots of potato cultivar under different levels of Cadmium stress i.e., Control= 0mg per kg, T1= 1mg per kg and T2= 5mg per kg).

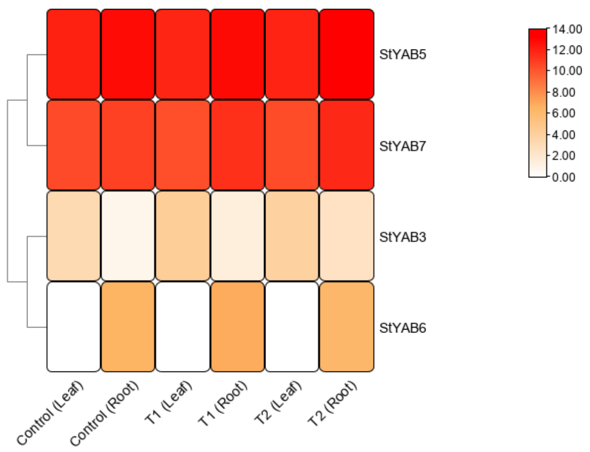

Figure S7:

Heat map shows the expression profile of the *StYAB* genes in Dormant and Non- Dormant potato cultivar.

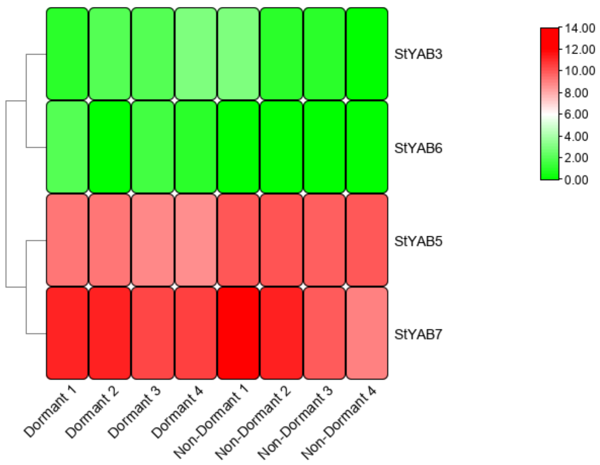

Figure S8:

Heat map shows the expression profile of the *StYAB* genes in leaves of four different potato cultivars under drought stress.

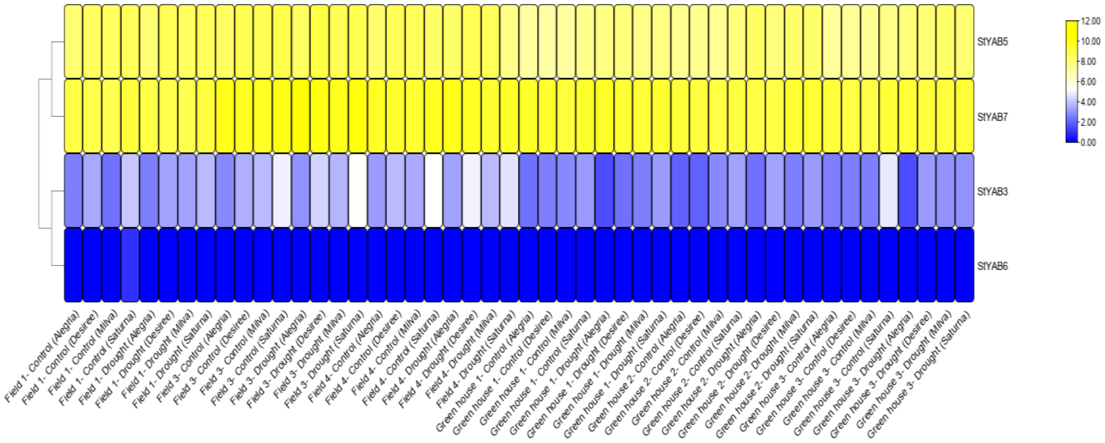

Figure S9:

Heat map shows the expression profile of the *StYAB* genes in petiole of potato cultivars under different photoperiod.

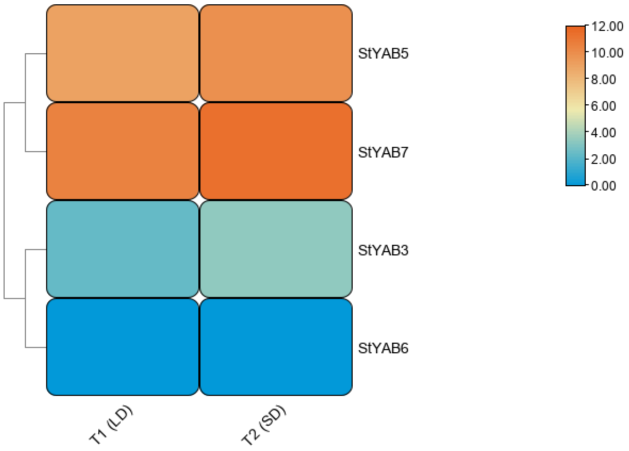

Figure S10:

Heat map shows the expression profile of the *StYAB* genes in phloem associated cells of petiole and lower stem of potato cultivar.

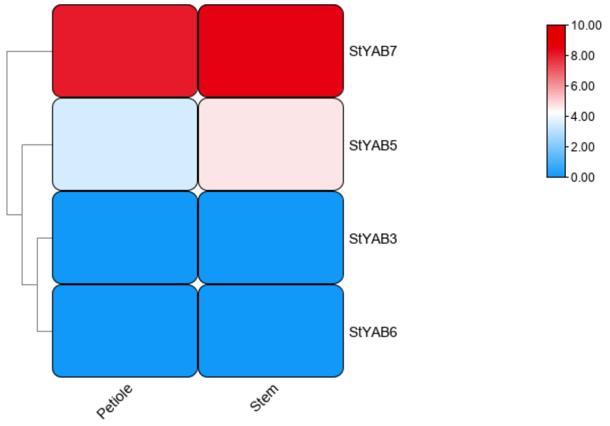

Figure S11:

Heat map shows the expression profile of the *StYAB* genes in tubers of two potato cultivars (Phytophthora Infestans resistant and susceptible) after different time of inoculating infection in both cultivars (0 hours, 24hours, and 48 hours).

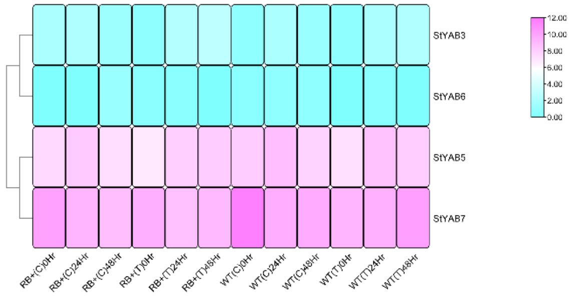

Supplement: Supplementary file 1 [file genes-14-00824-s001.zip › genes-2284668-Supplementary.pdf]
